# Allogeneic Hematopoietic Stem Cell Transplantation as a Therapeutic Approach for Hereditary Diseases

**DOI:** 10.3390/biomedicines13122903

**Published:** 2025-11-27

**Authors:** Sabina Nagieva, Svetlana Smirnikhina

**Affiliations:** Research Centre for Medical Genetics, 115522 Moscow, Russia

**Keywords:** allogeneic HSCT, hematopoietic stem cells, hemoglobinopathies, hereditary immunodeficiencies, lysosomal storage diseases (LSDs)

## Abstract

**Background/Objectives:** Allogeneic hematopoietic stem cell transplantation (allo-HSCT) is an established therapy for a range of hereditary disorders, including hemoglobinopathies, primary immunodeficiencies, and lysosomal storage diseases. Despite its long-standing use, rapid developments in donor availability, conditioning strategies, and supportive care have significantly broadened and refined its clinical application. This review combines recent evidence to clarify how these advances redefine current indications and therapeutic expectations. **Methods:** We critically analyze contemporary clinical data with a focus on elements that have undergone meaningful evolution—donor selection algorithms, conditioning intensity, graft manipulation, and post-transplant management. Comparative outcomes across major hereditary disease groups were examined to identify emerging trends in efficacy and safety. **Results:** The analysis highlights several novel shifts: expanding eligibility due to improved donor options, increasing reliance on reduced-toxicity regimens, and enhanced understanding of the mechanistic basis for hematologic, immunologic, and metabolic correction. These developments collectively improve survival and functional outcomes across diverse hereditary disorders. **Conclusions:** allo-HSCT remains a key therapeutic strategy for selected hereditary diseases, offering durable hematologic and metabolic correction. The prospective development of gene-addition and genome-editing therapies creates opportunities to complement—or in some cases replace—allo-HSCT, supporting the emergence of more personalized treatment approaches.

## 1. Introduction

Hematopoietic Stem Cell Transplantation (HSCT) is a therapeutic approach involving the replacement of hematopoietic stem cells to restore the functions of the human hematopoietic and immune systems. HSCT also represents a potentially effective strategy for enzyme replacement, providing the deficient protein in patients [1]. Currently, this method is employed not only for the treatment of hematologic and oncologic disorders but also for both symptomatic and pathogenetic therapy of hereditary diseases.

Genetic disorders impose a considerable global health burden, as a substantial proportion of births worldwide are affected by conditions with a hereditary or partially hereditary basis. Many of these conditions, such as hemoglobinopathies, primary immunodeficiencies, several inborn errors of metabolism and some other diseases, carry high morbidity and mortality. Given this enormous and global prevalence, allo-HSCT offers a uniquely powerful therapeutic strategy by providing long-term restoration of hematopoietic, immune, and metabolic function in patients with hereditary diseases.

**Figure 1:**
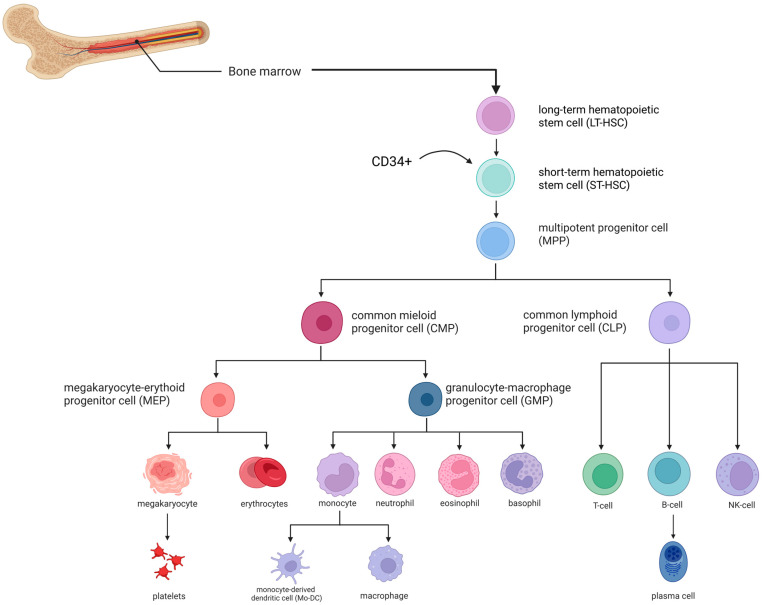
Hematopoiesis scheme [2,3]. The arrow indicates the population of CD34^+^ hematopoietic stem and progenitor cells illustrated in the early stages of the differentiation hierarchy. Image created using BioRender.com. Nagieva S. (2025) https://app.biorender.com/illustrations/68626b5bd8619352b0966ea3 (accessed on 1 October 2025).

A thorough understanding of the mechanisms underlying normal hematopoiesis is crucial for evaluating the efficacy and safety of hematopoietic stem cell transplantation aimed at restoring blood cell production in hereditary disorders. The accompanying scheme illustrates the organization of the hematopoietic system, demonstrating the differentiation of cells from the stem cell level to mature forms, which participate in the regeneration of hematopoiesis following transplantation (Figure 1).

Depending on the source of hematopoietic stem cells (HSCs) and the donor type, HSCT can be classified into several main types:

Allogeneic Hematopoietic Stem Cell Transplantation (allo-HSCT) involves the use of donor-derived stem cells. This method enables the restoration of normal hematopoiesis through the engraftment and proliferation of donor hematopoietic progenitor cells. Suitable donors for allo-HSCT include related HLA-identical, related HLA-mismatched or haploidentical, unrelated HLA-identical, and unrelated HLA-mismatched individuals. According to the literature data, umbilical cord blood can also serve as a graft source in a limited number of cases, due to the restricted cell quantity in a single cord blood unit and specific engraftment characteristics [4,5].

Allo-HSCT allows for maximal replacement of the patient’s pathological hematopoietic and immune cell clones [6]. Another advantage of this approach is the possibility of utilizing donor material when autologous HSCT is not feasible. Additionally, allo-HSCT confers a graft-versus-leukemia (GVL) effect, whereby donor immune cells recognize and eliminate residual malignant cells, thereby reducing the risk of leukemia relapse [7]. Despite its high efficacy in treating malignant and certain hereditary disorders, the method is associated with limitations, including immunologic and toxic complications, which must be considered when selecting a donor [8].

Autologous Hematopoietic Stem Cell Transplantation (auto-HSCT) uses the patient’s own stem cells for transplantation. This approach is primarily applied in the treatment of oncologic diseases in which the hematopoietic system itself is not affected. In such cases, auto-HSCT serves to restore hematopoietic function following intensive chemotherapy [9]. Patient-derived HSCs are collected, cryopreserved, and later reinfused. The main advantages of auto-HSCT include the absence of graft-versus-host disease (GVHD), as the transplanted cells are autologous.

## 2. Historical Background

The first attempt to restore hematopoietic function using bone marrow transfusion illustrates how the field progressed from empirical experimentation to a biologically informed, precision-based therapy. In that study in 1939, 43 daily transfusions and intravenous sternal bone marrow infusions were performed on a patient with aplastic anemia [10]. Although the procedure did not achieve the expected increase in leukocyte and platelet counts, it shifted thinking toward the hematopoietic system as a transplantable, regenerable organ long before the mechanisms of stem cell biology were understood.

In 1957, Edward Donnall Thomas and colleagues described the transplantation of HSCs from a healthy donor to patients with oncologic diseases who had undergone radio- and chemotherapy. At that time, all patients succumbed within 100 days due to severe graft-versus-host effects [11]. Only in 1979, following the study of histocompatibility systems, were the first successful outcomes of allogeneic HSCT reported, with 50% of patients with acute lymphoblastic leukemia achieving remission [12].

HSCT for hereditary diseases was first applied in 1968 to treat a five-month-old child with severe combined immunodeficiency (SCID), using the bone marrow of the patient’s sister as the donor source [13]. This procedure introduced another breakthrough: that HSCT could be curative even without cytotoxic conditioning, provided the recipient had no functional immunity capable of rejecting donor cells. This revealed that the success of HSCT depends not only on donor compatibility but also on the underlying pathophysiology of the disease. It opened the door to treating hereditary disorders, demonstrating that transplantation could replace entire defective cellular lineages, not just rescue hematopoietic recovery after cancer therapy. The expansion of HSCT in the 1970s–1980s to metabolic diseases [14] and hemoglobinopathies [15] represented a shift from reactive treatment of malignancy toward proactive correction of genetic disorders. This period marked the conceptual foundation of HSCT as a platform for disease modification—an idea that directly paved the way for today’s gene-therapy–enhanced autologous approaches.

## 3. Allogeneic Hematopoietic Stem Cell Transplantation for the Treatment of Various Groups of Hereditary Disorders (Table 1)

### 3.1. Allogeneic HSCT for the Treatment of Hemoglobinopathies

Hemoglobinopathies are a group of inherited disorders characterized by structural defects or impaired synthesis of hemoglobin. These pathologies arise from biallelic pathogenic variants in the genes encoding globin chains, leading to disrupted erythrocyte function and clinical manifestations including anemia, hypoxia, and hemolysis. Structural hemoglobin abnormalities include sickle cell disease (SCD), while conditions associated with impaired globin chain synthesis include alpha- and beta-thalassemia [16].

**Table 1:** Clinical outcomes and key limitations of Allo-HSCT in major hereditary disorders. HLA—Human Leukocyte Antigen, GVHD—Graft-Versus-Host Disease, SCID—Severe Combined Immunodeficiency, PID—Primary Immunodeficiency, MPS I—Mucopolysaccharidosis Type I, ERT—Enzyme Replacement Therapy, HSCT—Hematopoietic Stem Cell Transplantation.

Disorder	Survival Outcomes	Key Limitations/Complications
Sickle Cell Disease	High survival with HLA-identical sibling donors; best outcomes in early childhood.	Graft rejection, acute/chronic GVHD, transplant-related toxicity; reduced benefit in adults with organ damage.
β-Thalassemia	Highest survival in patients transplanted before adolescence; reduced survival in adults with advanced disease.	Graft failure or recurrence of native hematopoiesis; GVHD, alloimmunization, and iron overload increasing risk.
Primary Immunodeficiencies	Excellent outcomes in infants with SCID; variable survival in broader PID cohorts.	GVHD, graft rejection, infectious complications; long-term endocrine, fertility, and malignancy risks.
MPS I	Improved cognitive outcomes when transplanted pre-symptomatically.	Limited effect on established skeletal or cardiac pathology; insufficient neurological benefit if delayed.
Krabbe Disease	Better outcomes when HSCT is performed in the neonatal period; variable benefit in later-onset forms.	Persistent neurodevelopmental deficits; high dependence on timing; notable mortality in some cohorts.
Niemann–Pick Type B	Potential improvement in hepatic and pulmonary function.	Minimal impact on neurological disease; higher risk profile compared to ERT; use is highly restricted.
Metachromatic Leukodystrophy	Survival depends on disease stage at transplantation, with best results in early or pre-symptomatic cases.	Limited efficacy once neurodegeneration is established; progression may continue despite HSCT.

Sickle Cell Disease is defined by the presence of at least one HbS allele (p.(Glu6Val) variant in the *HBB* gene) along with a second pathogenic variant in *HBB*, resulting in abnormal hemoglobin polymerization and erythrocyte deformation. The most common form is the homozygous p.Glu6Val (Hb S/S) variant. Major clinical manifestations include pallor, jaundice, and chronic pain due to vaso-occlusive episodes in internal organs, hemolytic anemia, and splenomegaly. Globally, approximately 4 million individuals are affected by SCD, with most cases in Russia concentrated in southern regions near endemic areas [17,18].

Allogeneic HSCT from a healthy donor can replace the patient’s defective hematopoietic cell population with cells producing normal hemoglobin A (HbA) instead of mutant HbS, significantly improving survival. In an international study of 1000 patients with SCD who received allo-HSCT from HLA-identical siblings, the median age at transplantation was 9 years. Optimal outcomes were observed in younger patients: among children under 5 years, five-year overall survival (OS) reached 99%, and event-free survival (EFS) 96%. In contrast, patients over 15 years had OS and EFS of 88% and 84%, respectively. Key factors associated with transplant failure included older age, use of peripheral blood rather than bone marrow as the graft source, and outdated conditioning regimens. In the overall cohort, graft rejection occurred in 23 patients, and 70 deaths were reported, mainly due to infections, graft-versus-host disease, and therapy-related toxicity. Despite this, five-year OS and EFS for the entire cohort were 92.9% and 91.4%, respectively. Transplantation in childhood demonstrates the greatest potential for life extension and durable remission, whereas efficacy in adults, particularly with organ involvement, is limited, and life expectancy remains below the general population [19].

A single-center study of allo-HSCT in patients with congenital hemoglobinopathies, including SCD, included 38 children (4 with SCD) with a median age of 8 years at transplantation. The use of HLA-identical sibling donors and pharmacokinetically tailored busulfan-based conditioning achieved primary graft engraftment in 92.5% of cases. Five-year OS and EFS were 90.5% and 81.7%, respectively. Six patients required retransplantation, while acute and chronic GVHD affected approximately one-fifth of patients, alongside a single case of hepatic veno-occlusive disease [20]. Other risks include infertility and endocrine disorders due to aggressive conditioning regimens and subsequent immunosuppression [21].

The development of autologous HSCT based on gene-replacement therapy or genome-editing technologies such as CRISPR-Cas9 offers a promising approach for curative treatment of SCD without the risk of graft rejection or GVHD. Over the past 5–10 years, approximately 50–150 HSCT procedures for SCD have been performed annually worldwide, with 30–80 procedures per year in the United States. Although relatively rare, the number of these transplants is gradually increasing due to expanded patient selection criteria, the use of haploidentical and alternative donors, and improved conditioning regimens [22,23].

Beta-Thalassemia is also caused by pathogenic variants in *HBB*, resulting in impaired or absent β-globin chain synthesis. Excess α-chains aggregate, damaging erythrocytes and causing hemolysis. Phenotypic heterogeneity depends on the type and combination of pathogenic variants. Three main forms are recognized: major, intermediate, and minor. Beta-thalassemia major manifests in early childhood with severe anemia, hepatosplenomegaly, craniofacial skeletal deformities, and requires regular transfusions. Intermediate forms are milder and may not require regular transfusions but are prone to chronic anemia and iron overload complications. Minor forms are often asymptomatic or present with mild microcytic hypochromic anemia and are usually identified during screening or family studies.

Following donor HSC transplantation, engraftment in the bone marrow initiates normal hematopoiesis, gradually displacing defective erythropoietic cells. This restores full hemoglobin synthesis, corrects α-/β-globin chain imbalance, improves erythrocyte maturation, and reduces hemolysis, eliminating the need for regular transfusions and significantly improving quality of life [24]. Data from the international registry of beta-thalassemia patients who underwent allo-HSCT show overall survival of approximately 90%, with children and adolescents under 14 treated at an earlier age achieving up to 96% survival. Adults with more advanced disease and extensive clinical manifestations had survival rates around 80% [25].

Graft rejection or recurrence of recipient hematopoiesis occurs in 10–15% of cases [26], influenced by factors such as inadequate immunosuppression or conditioning, particularly in patients with high erythropoietic activity. Alloimmunization from multiple prior transfusions increases immune responses against donor cells, and severe iron overload exerts toxic effects on bone marrow and other organs, reducing transplant tolerance [27]. Use of peripheral blood stem cells (PBSCs) rather than bone marrow is associated with more pronounced immune reactions, including acute and chronic GVHD, further increasing rejection risk [28]. According to the American Society for Blood and Marrow Transplantation, 15 patients (47%) with primary and 14 (46.6%) with secondary graft failure underwent a second allo-HSCT among 62 patients with failed HSCTs out of 513 procedures [29]. Caocci et al. reported that a successful first HSCT ensures long-term survival comparable to conservative management while providing transfusion independence and maintaining good quality of life in the long term [30].

### 3.2. Allogeneic HSCT for the Treatment of Inherited Primary Immunodeficiencies

Inherited primary immunodeficiencies (PIDs) are a group of monogenic disorders characterized by defects in one or more components of the immune system. These conditions manifest as increased susceptibility to recurrent infections, autoimmune and autoinflammatory diseases, or malignancies. According to the 2022 update from the International Union of Immunological Societies, ten main groups of PIDs have been classified based on molecular-genetic and clinical-immunological characteristics [31]:

1. Combined immunodeficiencies
2. Combined immunodeficiencies with additional phenotypic features
3. B-cell deficiencies, agammaglobulinemia, or hypogammaglobulinemia
4. Immune dysregulation disorders
5. Congenital defects of phagocytes (quantitative or functional)
6. Defects of innate immunity
7. Autoinflammatory diseases
8. Complement system deficiencies
9. Bone marrow functional disorders
10. Phenocopies of primary immunodeficiencies

Allogeneic HSCT has demonstrated high efficacy as a primary therapeutic approach for patients with PIDs. In these patients, autologous hematopoietic stem cells are unable to produce fully functional lymphocytes, rendering them more vulnerable to infections and autoimmune manifestations. Following transplantation, the immune system is reconstituted from healthy donor cells. Timely allo-HSCT is critical for optimal outcomes in PIDs, with the highest survival rates observed when transplantation occurs in infancy, prior to the onset of severe infections or irreversible organ damage. For example, infants under 3.5 months with severe combined immunodeficiency achieved a five-year overall survival (OS) of 94% [32].

A retrospective analysis of 135 transplants conducted in Australia and New Zealand for patients with SCID, Wiskott-Aldrich syndrome, and chronic granulomatous disease reported a five-year OS of 72%. Disease-specific survival rates were 70%, 81%, and 69%, respectively. The study found no statistically significant effect of donor type (related vs. unrelated) or graft source on OS or transplant-related mortality. Notably, the use of unrelated HLA-identical cord blood produced outcomes comparable to unrelated HLA-identical bone marrow transplantation [33].

In a 32-year study at IRCCS Istituto Giannina Gaslini (Italy), outcomes of 67 transplants in children with congenital immunodeficiencies were analyzed. Five-year OS was 83.4%, with a median patient age of 2.5 years and median follow-up of 4 years. Graft rejection—both acute (immediately or within the first few weeks) and chronic (months post-transplant)—occurred in only 10.9% of cases. Acute graft-versus-host disease was observed in 18.7% of patients, less frequently in haploidentical transplants. The authors reported improved survival and reduced complications over time with the use of TCRαβ/CD19 depletion [34].

Allogeneic HSCT with TCRαβ/CD19 depletion is particularly useful when urgent transplantation is required from a related HLA-mismatched (haploidentical) donor. After apheresis and cell washing, cells are labeled and incubated with biotin-conjugated anti-TCRαβ antibodies. Following additional washing, paramagnetic microspheres conjugated with anti-biotin and anti-CD19 antibodies are added to selectively bind TCRαβ- and CD19-positive cells. The suspension is then processed through a magnetic column, retaining labeled cells while unlabeled cells pass through. The final graft is enriched in CD34^+^ hematopoietic stem cells, TCRγδ T cells, and NK cells, with minimal residual TCRαβ and B cells (CD19) [35,36].

However, allogeneic HSCT is not suitable for all patients. It is indicated for severe PIDs with high risk of mortality without transplantation. Donor availability is a critical factor. In the absence of a related donor, or in patients with significant somatic complications, transplantation carries a high risk of adverse events [37,38], including fungal infections of the skin, liver, or gastrointestinal tract. Early post-transplant infections are particularly concerning under immunosuppressive therapy used to prevent graft rejection. Long-term risks include endocrine disorders, infertility, and secondary malignancies [39].

Despite potential risks, HSCT markedly improves prognosis for most patients with severe PIDs. Early diagnosis, timely transplantation, and the availability of a compatible donor enable near-normal life expectancy and quality of life. Contemporary data indicate substantial improvements in survival: in many forms of PID, five-year OS exceeds 70–94%, especially when transplantation occurs in infancy before the development of infectious complications.

### 3.3. Allogeneic HSCT for the Treatment of Lysosomal Storage Disorders

Lysosomal storage disorders (LSDs) are inherited diseases caused by defects in lysosomal enzymes, leading to abnormal accumulation of corresponding substrates. As a result, LSDs are characterized by multisystemic dysfunctions affecting various organs. Allogeneic HSCT aims to replace the patient’s defective cells with healthy donor-derived hematopoietic stem cells capable of producing the necessary lysosomal enzymes.

Recent clinical studies consistently demonstrate that the degree of donor hematopoietic chimerism is a key determinant of metabolic correction for lysosomal storage disorders. Full or near-full donor chimerism is strongly associated with normalization of lysosomal enzyme activity and improved biochemical markers, including reduction of accumulated substrates [40]. Patients achieving complete donor engraftment show the most effective metabolic response, particularly in mucopolysaccharidosis type I and related disorders, whereas mixed chimerism often results in low enzyme levels and incomplete substrate clearance [41]. Long-term follow-up indicates that stable donor chimerism—especially from a non-carrier donor—supports sustained enzyme correction, while declining chimerism may lead to partial biochemical relapse [42]. Collectively, these findings underscore that both the magnitude and lineage distribution of donor chimerism are critical parameters for achieving durable metabolic correction after HSCT.

To date, allo-HSCT has demonstrated efficacy in the treatment of several LSDs, including mucopolysaccharidosis type I (MPS I, Hurler syndrome), types II, IV, and VI, Krabbe disease, adrenoleukodystrophy, metachromatic leukodystrophy, Gaucher disease, and others (Table 2).

Allogeneic HSCT for mucopolysaccharidosis type I (MPS I) significantly improves cognitive function. However, its ability to slow neurodegeneration is most evident when performed before neurological symptoms appear, highlighting the need for early intervention [43]. The procedure does not reverse established skeletal deformities or cardiac valve defects. For optimal outcomes, HSCT is recommended in combination with enzyme replacement therapy (ERT) specifically developed for MPS I. Based on a European consensus process for the management of MPS I, it is recommended to initiate ERT immediately after diagnosis while awaiting allo-HSCT, ideally before the age of 2.5 years [44].

Gene therapy approaches are also being developed for MPS I, aimed at delivering a functional copy of the *IDUA* gene. One of the leading candidates, OTL-203, uses autologous hematopoietic stem cells modified with a lentiviral vector and has demonstrated promising clinical benefits, including cognitive and somatic improvement.

For Krabbe disease, allo-HSCT is applied in late infantile (2–4 years) and juvenile (after 6 years) forms. Outcomes vary depending on the timing of transplantation and disease severity at the time of the procedure. In one patient cohort, post-transplantation motor deficits persisted into adolescence, while another cohort exhibited no disease manifestations, with 25% mortality over 15 years [45]. Transplantation within the first month of life in asymptomatic carriers of pathogenic GALC variants resulted in normal enzyme activity. However, the effect was not permanent: during follow-up from 4 months to 6 years, cognitive and motor impairments emerged [46]. Early identification of Krabbe disease via preclinical or neonatal screening can substantially improve quality of life and survival when HSCT is performed within the first 14 days of life [47].

Allogeneic HSCT for Niemann-Pick disease type B offers potential benefits for hepatic and pulmonary manifestations by normalizing sphingomyelinase activity [48,49]. Its efficacy is limited regarding neurological symptoms, and CNS involvement must be carefully considered when selecting therapy. ERT with olipudase alfa (Xenpozyme) is currently preferred and safer, particularly for systemic manifestations without severe CNS involvement. Allo-HSCT is considered an alternative when ERT is unavailable or within research protocols [50].

For metachromatic leukodystrophy, allo-HSCT is used in late infantile (6 months–4 years) and early juvenile (4–6 years) forms, and occasionally in adult-onset disease (≥16 years) to address primary CNS manifestations. Five-year overall survival ranges from 42% to 72%, without clear differences among subtypes [51]. Libmeldy (atidarsagene autotemcel), an autologous HSCT using gene-modified hematopoietic stem cells, is approved in the US and Europe for presymptomatic late infantile and early juvenile patients with preserved ambulation and before cognitive decline [52]. Lentiviral vector-based autologous HSCT in preclinical studies demonstrated a fourfold increase in ARSA gene expression compared with the approved product [53].

Allogeneic HSCT remains a pathogenetically justified option for several lysosomal storage disorders, particularly in patients with systemic involvement and when performed early. However, transplant-related risks and limited impact on neurological outcomes, especially with CNS disease, constrain its wider use. Today, HSCT is mainly considered as an adjunct or alternative to enzyme replacement therapy.

## 4. Procedure for Allogeneic Hematopoietic Stem Cell Transplantation in the Treatment of Hereditary Disorders

To determine the necessity of allogeneic hematopoietic stem cell transplantation (allo-HSCT), a comprehensive assessment of clinical indications should be performed [54,55]:

- Confirm the patient’s diagnosis through molecular-genetic testing.
- Assess disease stage and severity, as HSCT is most effective in many disorders, such as lysosomal storage diseases (LSDs), when performed prior to the onset of neurological symptoms.
- Evaluate the patient’s somatic condition, since individuals with severe multi-organ failure may have contraindications to myeloablative conditioning and an increased risk of mortality.
- Consider the patient’s age and weight, as transplantation outcomes tend to be more favorable at younger ages.
- Review the availability of alternative therapies, since for certain hereditary disorders, enzyme replacement therapy (ERT) or gene therapy may represent the most effective treatment option.

According to the recommendations of the European Society for Blood and Marrow Transplantation (EBMT), indications for HSCT are divided into four main categories: standard of care (S), clinical option (CO), under development (D), and generally not recommended (GNR). For diseases classified as S, transplantation provides superior outcomes compared with alternative treatments. In the CO category, efficacy is recognized, but the small patient population limits the availability of robust comparative data. Indications within the D category are currently investigational, and transplantation should be performed within scientific protocols. In situations classified as GNR, transplantation is generally considered inappropriate [56,57].

## 5. Steps of Allogeneic HSCT (Figure 2)

1. **Donor Selection and HLA Typing.** Determining indications for HSCT, HLA typing is performed for the patient and their immediate relatives (parents and siblings). Selection of the donor and graft source must consider several clinical factors, including disease type and stage, remission status, and transplantation urgency. While an HLA-identical related donor (sibling) remains preferred, survival outcomes are comparable when using an HLA-matched unrelated donor. In the absence of a fully matched related or unrelated donor, haploidentical transplantation from a related donor may be considered. Recent data suggest that, under certain conditions, haploidentical HSCT can achieve comparable efficacy and graft-versus-host disease risk to fully matched donor transplantation.
2. **Graft Collection and Preparation.** Peripheral blood stem cells (PBSC) have increasingly become the preferred source of hematopoietic stem cells (HSCs) over bone marrow. To maximize the CD34^+^ stem cell pool, donors are often mobilized with recombinant granulocyte colony-stimulating factor (G-CSF) or, less commonly, plerixafor, a reversible CXCR4 antagonist, which stimulates leukopoiesis and mobilizes HSCs into peripheral blood. Collection is performed via apheresis. In some centers, graft manipulation is performed, such as T-cell depletion through TCRαβ/CD19 elimination, as previously described [58].
3. **Recipient Conditioning.** After comprehensive evaluation, indication confirmation, and planning, the recipient undergoes conditioning. Conditioning involves chemotherapy and/or total body irradiation (TBI) to induce myelo- and immunosuppression. This step creates bone marrow niches for donor HSC engraftment and prevents graft-versus-host reactions. Conditioning regimens are classified as myeloablative, non-myeloablative, reduced-intensity, and immunoablative.

- Myeloablative conditioning employs high-dose chemotherapy and/or TBI, almost completely eradicating the recipient’s bone marrow; this is standard for hemoglobinopathies.
- Non-myeloablative conditioning uses minimal chemotherapy doses, preserving endogenous hematopoiesis and relying partly on the graft-versus-leukemia effect. This approach is rare and mainly for non-malignant diseases or patient’s intolerant to toxic regimens.
- Reduced-intensity conditioning partially suppresses bone marrow while significantly reducing immune activity, suitable for patients with comorbidities or older age.
- Immunoablative conditioning primarily suppresses a hyperactive or pathological immune system, partially preserving marrow function. This regimen is widely used in non-malignant disorders involving immune overactivation, such as aplastic anemia.

Latest reports emphasize that optimizing conditioning protocols is critical for improving the safety and efficacy of allogeneic HSCT in non-malignant disorders. Traditional myeloablative conditioning is associated with substantial toxicity, leading to a transition toward reduced-toxicity and reduced-intensity approaches, particularly for pediatric patients and those with significant comorbidities. Treosulfan-based regimens have demonstrated favorable toxicity profiles and are increasingly recommended for primary immunodeficiencies [59]. In transfusion-dependent β-thalassemia, protocols combining pre-transplant immunosuppression with modified myeloablative conditioning have improved engraftment stability, reduced mixed chimerism, and achieved high overall survival and transfusion-free survival rates [60]. Additionally, non-myeloablative conditioning strategies have shown effectiveness in second HSCT after graft failure, achieving durable engraftment with comparatively lower toxicity [61,62]. These advances highlight the need for ongoing refinement of conditioning regimens personalized to non-malignant diseases to minimize late complications while maintaining reliable donor cell engraftment.

4. **Stem Cell Transplantation and Engraftment.** At this stage, the selected CD34^+^ cells are infused into the patient via a central venous catheter.
5. **Early Post-Transplant Period**. Engraftment typically occurs between 14 and 28 days [63,64]. During this period, colony-stimulating factors are administered, and additional transfusions may be provided to support hematopoiesis. To reduce graft rejection and acute GVHD risk, a combination of chemotherapy and immunosuppressive therapy is initiated. Successful haploidentical transplantation is enhanced by the use of antithymocyte globulin during conditioning, post-transplant cyclophosphamide for in vivo T-cell depletion, or a combination thereof [65,66]. After allo-HSCT, the emergence of immune tolerance can be tracked through T cells, early NK-cell reconstitution, and cytokine profiles. Higher levels of donor-derived regulatory T cells (CD4^+^CD25^+^FoxP3^+^) are linked to reduced GVHD risk and better immune control. In the early post-transplant phase, changes in cytokines such as IL-10, TGF-β, and others reflect the balance between immune activation and regulation [67,68]. Supportive measures include transfusions and G-CSF to stimulate leukopoiesis. In the context of chemotherapy-induced aplasia, continuous microbiological monitoring is critical, requiring prophylactic antimicrobial therapy, including antifungal agents, as well as timely initiation of antibacterial and antiviral treatments. Bone marrow aspirates are performed to evaluate myelogram and chimerism. In cases of suboptimal chimerism, immunosuppressive therapy intensity is adjusted, or donor lymphocyte infusions (DLI) are administered when feasible [69].
6. **Long-Term Follow-Up**. Post-transplant follow-up involves monitoring for disease manifestations, chimerism status, signs of acute (≤100 days) and chronic (>100 days) GVHD, vaccination, functional recovery measures, and assessment of long-term prognosis and quality of life.

**Figure 2:**
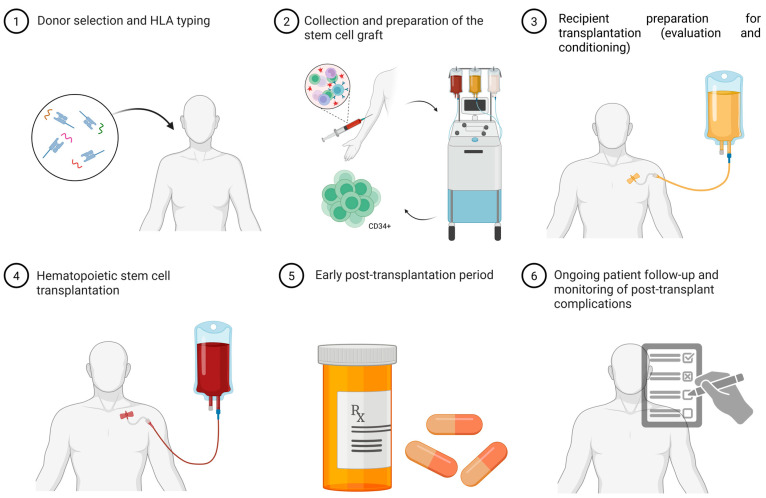
Allogeneic HSCT procedure steps. Descriptions for each step illustrated in the scheme are provided in the relevant section of the article. HLA typing—Human Leukocyte Antigen typing (molecular analysis of donor–recipient HLA genes to assess immunological compatibility). CD34*^+^* cells—hematopoietic stem and progenitor cells capable of self-renewal and multilineage differentiation. Image created using BioRender.com. Nagieva S. (2025) https://app.biorender.com/illustrations/686290e663aa6d29145612a3 (accessed on 1 October 2025).

## 6. Relevance of Allogeneic HSCT

Hematopoietic stem cell transplantation is a highly effective treatment for a range of severe hereditary disorders, including primary immunodeficiencies, hemoglobinopathies (such as beta-thalassemia and sickle cell disease), and lysosomal storage disorders. The success of therapy largely depends on the timing of transplantation. Early intervention, before the onset of significant clinical complications, substantially improves survival, enhances quality of life, and reduces the risk of disability. Therefore, the diagnosis of these disorders in the earliest days of life, at preclinical stages, is critical for timely initiation of treatment. Neonatal screening has become a key tool for such early detection and is actively implemented in healthcare systems in many developed countries.

In several countries worldwide, neonatal screening covers a broad spectrum of conditions potentially amenable to HSCT. In the United States, since 2018, mandatory screening includes multiple hereditary disorders, such as beta-thalassemia, sickle cell disease, severe combined immunodeficiency, and certain lysosomal storage disorders (MPS I, Pompe disease, adrenoleukodystrophy, among others) [70]. Expanded newborn screening panels are also in place in European countries, Japan, Israel, and Australia, encompassing metabolic disorders, immunodeficiencies, and hematologic pathologies [71]. In Russia, expanded neonatal screening including TREC/KREC quantification was introduced in 2023, leading to the diagnosis of 726 children in 2024, including 121 with primary immunodeficiencies, highlighting the program’s effectiveness and need for further expansion.

Recent progress in gene-therapy-based autologous HSCT provides curative alternatives without the risks of graft rejection or GVHD. Lentiviral-modified or CRISPR-edited autologous hematopoietic stem cells have shown high efficacy for β-thalassemia, SCD, MPS I, and metachromatic leukodystrophy, in some cases outperforming traditional allogeneic HSCT in safety profiles. By using a patient’s own hematopoietic stem cells genetically corrected ex vivo or via lentiviral vectors or CRISPR-based editing—these therapies eliminate the need for a matched donor and virtually remove the risks of graft-versus-host disease or graft rejection. Early diagnosis through neonatal screening is becoming even more critical, as timely access to both allogeneic and autologous gene-therapy HSCT prevents irreversible damage and reduces long-term reliance on supportive therapies.

## 7. Conclusions

Allogeneic hematopoietic stem cell transplantation continues to play a pivotal role in the treatment of severe hereditary disorders, remaining an effective method of disease-modifying therapy when performed in a timely manner. Clinical outcomes support the use of this approach in cases of early diagnosis and appropriate indications. However, strict patient selection criteria, procedural risks, and limited efficacy in the presence of severe central nervous system involvement remain challenges requiring further investigation. Current progress in gene-therapy-based autologous HSCT—using lentiviral vectors or genome editing - offers a promising alternative that avoids graft-versus-host disease and expands eligibility for patients lacking matched donors. However, the long-term durability and safety of gene-modified grafts require further study, particularly regarding insertional mutagenesis, immune dysregulation, and late organ toxicity. Optimal conditioning strategies that balance engraftment with reduced toxicity remain undefined, especially for infants and patients with comorbidities. Additionally, reliable biomarkers predicting treatment response and the ideal timing of intervention are still lacking. Future efforts should focus on refining low-toxicity conditioning, improving long-term safety of gene-modified cells, establishing optimal timing for intervention, and integrating newborn screening data to guide personalized, earlier treatment strategies. This direction of research is essential for maximizing clinical benefit and reducing lifelong treatment burdens.

## Figures and Tables

**Table 2:** Allogeneic HSCT in Lysosomal Storage Diseases. LSD—Lysosomal Storage Diseases, AR—Autosomal Recessive Inheritance, GVHD—Graft-versus-Host Disease, ERT—Enzyme Replacement Therapy, SRT—Substrate Reduction Therapy, HRT—Hormone Replacement Therapy, EMA—European Medicine Agency. *—with early allogeneic HSCT [43,44,45,46,47,48,49,50,51,52,53].

Lysosomal Storage Diseases Amenable to Allo-HSCT	Gene, Inheritance Type	Global Prevalence	Optimal Timing for Transplantation	5-Year Survival Rate *	Indications	Common Side Effects	Contraindications	Alternative Treatment Options	Worldwide Newborn Screening
MPS I (Hurler syndrome)	*IDUA*, AR	1:100,000	Before 2 years of age (prior to neurological symptoms)	70–80%	Severe form without marked CNS involvement	GVHD, infections	Skeletal deformities, heart valve defects, severe CNS damage	ERT (Laronidase, preferably combined with HSCT), symptomatic therapy	Yes
Krabbe disease	*GALC*, AR	1:100,000	Within 30 days of life (pre-symptomatic stage)	70–80%	Pre-symptomatic stage in late infantile or juvenile forms	GVHD, cognitive and motor dysfunction	Severe neurological symptoms in late diagnosis	Symptomatic therapy	Yes (USA)
Niemann-Pick disease, type B	*SMPD1*, AR	1:250,000	Before onset of neurological symptoms	No average data	Severe liver and lung involvement	GVHD, infections	Presence of neurological symptoms	ERT (Olipudase alfa)	Yes (EMA)
Metachromatic leukodystrophy	*ARSA*, AR	1:40,000–100,000	Pre-symptomatic or early symptomatic stages	57–74%	Late infantile or early juvenile forms (rarely adult form)	GVHD, infections, neurological complications	Advanced neurological impairment	Gene therapy (Atidarsagene autotemcel), symptomatic therapy	Yes (EMA)
Adrenoleukodystrophy	*ABCD1*, X-linked recessive	1:20,000 (males)	Before severe brain damage (Loes score < 9)	85–90%	Childhood cerebral form (highest efficacy)	GVHD, endocrine disorders	Severe cerebral involvement (Loes score >9)	Gene therapy (Elivaldogene autotemcel), symptomatic therapy (including HRT)	Yes (USA)
Gaucher disease	*GBA*, AR	1:50,000	Before onset of neurological symptoms	<60%	Visceral involvement	GVHD, infections	Severe neurological symptoms	ERT (Imiglucerase), SRT	Yes

## Data Availability

No new data were created or analyzed in this study.

## References

[1] Siddiqi F., Wolfe J.H. (2016). Stem Cell Therapy for the Central Nervous System in Lysosomal Storage Diseases. Hum. Gene Ther..

[2] Cheng H., Zheng Z., Cheng T. (2020). New Paradigms on Hematopoietic Stem Cell Differentiation. Protein Cell.

[3] Zhang P., Zhang C., Li J., Han J., Liu X., Yang H. (2019). The Physical Microenvironment of Hematopoietic Stem Cells and Its Emerging Roles in Engineering Applications. Stem Cell Res. Ther..

[4] Xu L.-P., Huang X.-J. (2011). Current Status and Development of Hematopoietic Stem Cell Transplantation in China: A Report from Chinese Hematopoietic Stem Cell Transplantation Register Group. Chin. Med. J. (Engl.).

[5] Lv M., Huang X.-J. (2012). Allogeneic Hematopoietic Stem Cell Transplantation in China: Where We Are and Where to Go. J. Hematol. Oncol..

[6] Chapman M.S., Wilk C.M., Boettcher S., Mitchell E., Dawson K., Williams N., Müller J., Kovtonyuk L., Jung H., Caiado F. (2024). Clonal Dynamics after Allogeneic Haematopoietic Cell Transplantation. Nature.

[7] Kolb H.-J. (2008). Graft-Versus-Leukemia Effects of Transplantation and Donor Lymphocytes. Blood.

[8] Tomblyn M., Chiller T., Einsele H., Gress R., Sepkowitz K., Storek J., Wingard J.R., Young J.-A.H., Boeckh M.A. (2009). Guidelines for Preventing Infectious Complications Among Hematopoietic Cell Transplantation Recipients: A Global Perspective. Biol. Blood Marrow Transplant..

[9] Copelan E.A. (2006). Hematopoietic Stem-Cell Transplantation. N. Engl. J. Med..

[10] Osgood E.E., Riddle M.C., Mathews T.J. (1939). Aplastic Anemia Treated with Daily Transfusions and Intravenous Marrow: Case Report. Ann. Intern. Med..

[11] Thomas E.D., Lochte H.L., Lu W.C., Ferrebee J.W. (1957). Intravenous Infusion of Bone Marrow in Patients Receiving Radiation and Chemotherapy. N. Engl. J. Med..

[12] Thomas E.D., Buckner C.D., Clift R.A., Fefer A., Johnson F.L., Neiman P.E., Sale G.E., Sanders J.E., Singer J.W., Shulman H. (1979). Marrow Transplantation for Acute Nonlymphoblastic Leukemia in First Remission. N. Engl. J. Med..

[13] Gatti R., Meuwissen H., Allen H., Hong R., Good R. (1968). Immunological Reconstitution of Sex-Linked Lymphopenic Immunological Deficiency. Lancet.

[14] Hobbs J.R., Hugh-Jones K., Barrett A.J., Byrom N., Chambers D., Henry K., James D.C., Lucas C.F., Rogers T.R., Benson P.F. (1981). Reversal of Clinical Features of Hurler’s Disease and Biochemical Improvement after Treatment by Bone-Marrow Transplantation. Lancet.

[15] Johnson F.L., Look A.T., Gockerman J., Ruggiero M.R., Dalla-Pozza L., Billings F.T. (1984). Bone-Marrow Transplantation in a Patient with Sickle-Cell Anemia. N. Engl. J. Med..

[16] Harteveld C.L., Achour A., Arkesteijn S.J.G., ter Huurne J., Verschuren M., Bhagwandien-Bisoen S., Schaap R., Vijfhuizen L., El Idrissi H., Koopmann T.T. (2022). The Hemoglobinopathies, Molecular Disease Mechanisms and Diagnostics. Int. J. Lab. Hematol..

[17] Isah I.Z., Udomah F.P., Erhabor O., Aghedo F., Uko E.K., Okwesili A.N., Buhari H.A., Onuigwe F.U., Abdulrahaman Y., Adamu M. (2013). Foetal Haemoglobin Levels in Sickle Cell Disease (SCD) Patients in Sokoto, Nigeria. Br. J. Med. Health Sci..

[18] Rumyantsev A.G., Tokarev Y.N., Smetanina N.S., Maksimov V.A. (2023). Gemoglobinopatii i Talassemicheskie Sindromy [Hemoglobinopathies and Thalassemic Syndromes].

[19] Gluckman E., Cappelli B., Bernaudin F., Labopin M., Volt F., Carreras J., Simões B.P., Ferster A., Dupont S., de la Fuente J. (2017). Sickle Cell Disease: An International Survey of Results of HLA-Identical Sibling Hematopoietic Stem Cell Transplantation. Blood.

[20] Zaidman I., Rowe J.M., Khalil A., Ben-Arush M., Elhasid R. (2016). Allogeneic Stem Cell Transplantation in Congenital Hemoglobinopathies Using a Tailored Busulfan-Based Conditioning Regimen: Single-Center Experience. Biol. Blood Marrow Transplant..

[21] Kanter J., Liem R.I., Bernaudin F., Bolaños-Meade J., Fitzhugh C.D., Hankins J.S., Murad M.H., Panepinto J.A., Rondelli D., Shenoy S. (2021). American Society of Hematology 2021 Guidelines for Sickle Cell Disease: Stem Cell Transplantation. Blood Adv..

[22] Spellman S.R., Xu K., Oloyede T., Ahn K.W., Akhtar O., Bolon Y.-T., Broglie L., Bloomquist J., Bupp C., Chen M. (2025). Current Activity Trends and Outcomes in Hematopoietic Cell Transplantation and Cellular Therapy—A Report from the CIBMTR. Transplant Cell Ther..

[23] Jagasia M., Perales M.-A., Schroeder M.A., Ali H., Shah N.N., Chen Y.-B., Fazal S., Dawkins F.W., Arbushites M.C., Tian C. (2020). Ruxolitinib for the Treatment of Steroid-Refractory Acute GVHD (REACH1): A Multicenter, Open-Label Phase 2 Trial. Blood.

[24] Yousuf R., Jahan D., Sinha S., Haque M. (2023). Haematopoietic Stem Cell Transplantation in Thalassaemia Major: A Narrative Review. Adv. Hum. Biol..

[25] Baronciani D., Angelucci E., Potschger U., Gaziev J., Yesilipek A., Zecca M., Orofino M.G., Giardini C., Al-Ahmari A., Marktel S. (2016). Hemopoietic Stem Cell Transplantation in Thalassemia: A Report from the European Society for Blood and Bone Marrow Transplantation Hemoglobinopathy Registry, 2000–2010. Bone Marrow Transplant..

[26] Rahal I., Galambrun C., Bertrand Y., Garnier N., Paillard C., Frange P., Pondarré C., Dalle J.H., de Latour R.P., Michallet M. (2018). Late Effects after Hematopoietic Stem Cell Transplantation for β-Thalassemia Major: The French National Experience. Haematologica.

[27] Meissner B., Lang P., Bader P., Hoenig M., Müller I., Meisel R., Greil J., Sauer M.G., Metzler M., Corbacioglu S. (2024). Finding a Balance in Reduced Toxicity Hematopoietic Stem Cell Transplantation for Thalassemia: Role of Infused CD3+ Cell Count and Immunosuppression. Bone Marrow Transplant..

[28] Mulas O., Mola B., Caocci G., La Nasa G. (2022). Conditioning Regimens in Patients with β-Thalassemia Who Underwent Hematopoietic Stem Cell Transplantation: A Scoping Review. J. Clin. Med..

[29] Korula A., Pn N., Devasia A., Lakshmi K.M., Abraham A., Sindhuvi E., George B., Srivastava A., Mathews V. (2018). Second Hematopoietic Stem Cell Transplant for Thalassemia Major: Improved Clinical Outcomes with a Treosulfan-Based Conditioning Regimen. Biol. Blood Marrow Transplant..

[30] Caocci G., Orofino M.G., Vacca A., Piroddi A., Piras E., Addari M.C., Caria R., Pilia M.P., Origa R., Moi P. (2017). Long-Term Survival of Beta Thalassemia Major Patients Treated with Hematopoietic Stem Cell Transplantation Compared with Survival with Conventional Treatment. Am. J. Hematol..

[31] Tangye S.G., Al-Herz W., Bousfiha A., Cunningham-Rundles C., Franco J.L., Holland S.M., Klein C., Morio T., Oksenhendler E., Picard C. (2022). Human Inborn Errors of Immunity: 2022 Update on the Classification from the International Union of Immunological Societies Expert Committee. J. Clin. Immunol..

[32] Pai S.-Y., Logan B.R., Griffith L.M., Buckley R.H., Parrott R.E., Dvorak C.C., Kapoor N., Hanson I.C., Filipovich A.H., Jyonouchi S. (2014). Transplantation Outcomes for Severe Combined Immunodeficiency, 2000–2009. N. Engl. J. Med..

[33] Mitchell R., Nivison-Smith I., Anazodo A., Tiedemann K., Shaw P., Teague L., Fraser C., Carter T., Tapp H., Alvaro F. (2013). Outcomes of Hematopoietic Stem Cell Transplantation in Primary Immunodeficiency: A Report from the Australian and New Zealand Children’s Haematology Oncology Group and the Australasian Bone Marrow Transplant Recipient Registry. Biol. Blood Marrow Transplant..

[34] Dell’oRso G., Bagnasco F., Giardino S., Pierri F., Ferrando G., Di Martino D., Micalizzi C., Guardo D., Volpi S., Sabatini F. (2023). Hematopoietic Stem Cell Transplantation for Inborn Errors of Immunity: 30-Year Single-Center Experience. Front. Immunol..

[35] Pira G.L., Malaspina D., Girolami E., Biagini S., Cicchetti E., Conflitti G., Broglia M., Ceccarelli S., Lazzaro S., Pagliara D. (2016). Selective Depletion of αβ T Cells and B Cells for Human Leukocyte Antigen-Haploidentical Hematopoietic Stem Cell Transplantation: A Three-Year Follow-Up of Procedure Efficiency. Biol. Blood Marrow Transplant..

[36] Merli P., Algeri M., Galaverna F., Bertaina V., Lucarelli B., Boccieri E., Becilli M., Quagliarella F., Rosignoli C., Biagini S. (2024). TCRαβ/CD19 Cell–Depleted HLA-Haploidentical Transplantation to Treat Pediatric Acute Leukemia: Updated Final Analysis. Blood.

[37] Gavrilova T. (2019). Considerations for Hematopoietic Stem Cell Transplantation in Primary Immunodeficiency Disorders. World J. Transplant..

[38] Laberko A., Sultanova E., Gutovskaya E., Shipitsina I., Shelikhova L., Kurnikova E., Muzalevskii Y., Kazachenok A., Pershin D., Voronin K. (2019). Mismatched Related vs. Matched Unrelated Donors in TCRαβ/CD19-Depleted HSCT for Primary Immunodeficiencies. Blood.

[39] Ghimire S., Weber D., Mavin E., Wang X.N., Dickinson A.M., Holler E. (2017). Pathophysiology of GvHD and Other HSCT-Related Major Complications. Front. Immunol..

[40] Qu Y., Liu H., Wei L., Nie S., Ding W., Liu S., Liu H., Jiang H. (2022). The Outcome of Allogeneic Hematopoietic Stem Cell Transplantation from Different Donors in Recipients with Mucopolysaccharidosis. Front. Pediatr..

[41] Hampe C.S., Wesley J., Lund T.C., Orchard P.J., Polgreen L.E., Eisengart J.B., McLoon L.K., Cureoglu S., Schachern P., McIvor R.S. (2021). Mucopolysaccharidosis Type I: Current Treatments, Limitations, and Prospects for Improvement. Biomolecules.

[42] Gardin A., Castelle M., Pichard S., Cano A., Chabrol B., Piarroux J., Roubertie A., Nadjar Y., Guemann A., Tardieu M. (2023). Long Term Follow-Up after Haematopoietic Stem Cell Transplantation for Mucopolysaccharidosis Type I-H: A Retrospective Study of 51 Patients. Bone Marrow Transplant..

[43] Muenzer J., Wraith J.E., Clarke L.A., the International Consensus Panel on the Management and Treatment of Mucopolysaccharidosis I (2009). Mucopolysaccharidosis I: Management and Treatment Guidelines. Pediatrics.

[44] de Ru M.H., Boelens J.J., Das A.M., Jones S.A., van der Lee J.H., Mahlaoui N., Mengel E., Offringa M., O’Meara A., Parini R. (2011). Enzyme Replacement Therapy and/or Hematopoietic Stem Cell Transplantation at Diagnosis in Patients with Mucopolysaccharidosis Type I: Results of a European Consensus Procedure. Orphanet J. Rare Dis..

[45] Wright M.D., Poe M.D., DeRenzo A., Haldal S., Escolar M.L. (2017). Developmental Outcomes of Cord Blood Transplantation for Krabbe Disease. Neurology.

[46] Escolar M.L., Poe M.D., Provenzale J.M., Richards K.C., Allison J., Wood S., Wenger D.A., Pietryga D., Wall D., Champagne M. (2005). Transplantation of Umbilical-Cord Blood in Babies with Infantile Krabbe’s Disease. N. Engl. J. Med..

[47] Kwon J.M., Matern D., Kurtzberg J., Wrabetz L., Gelb M.H., Wenger D.A., Ficicioglu C., Waldman A.T., Burton B.K., Hopkins P.V. (2018). Consensus Guidelines for Newborn Screening, Diagnosis and Treatment of Infantile Krabbe Disease. Orphanet J. Rare Dis..

[48] Quarello P., Spada M., Porta F., Vassallo E., Timeus F., Fagioli F. (2018). Hematopoietic Stem Cell Transplantation in Niemann–Pick Disease Type B Monitored by Chitotriosidase Activity. Pediatr. Blood Cancer.

[49] Shah A.J., Kapoor N., Crooks G.M., Parkman R., Weinberg K.I., Wilson K., Kohn D.B. (2005). Successful Hematopoietic Stem Cell Transplantation for Niemann–Pick Disease Type B. Pediatrics.

[50] Lachmann R.H., Diaz G.A., Wasserstein M.P., Armstrong N.M., Yarramaneni A., Kim Y., Kumar M. (2023). Olipudase Alfa Enzyme Replacement Therapy for Acid Sphingomyelinase Deficiency (ASMD): Sustained Improvements in Clinical Outcomes after 6.5 Years of Treatment in Adults. Orphanet J. Rare Dis..

[51] Boucher A.A., Miller W., Shanley R., Ziegler R., Lund T., Raymond G., Orchard P.J. (2015). Long-Term Outcomes after Allogeneic Hematopoietic Stem Cell Transplantation for Metachromatic Leukodystrophy: The Largest Single-Institution Cohort Report. Orphanet J. Rare Dis..

[52] Wynn R.F., Wraith J.E., Mercer J., Frcpi A.O., Bsc K.T., Thornley M., Church H.J., Bigger B.W. (2009). Improved Metabolic Correction in Patients with Lysosomal Storage Disease Treated with Hematopoietic Stem Cell Transplant Compared with Enzyme Replacement Therapy. J. Pediatr..

[53] Tricoli L., Sase S., Hacker J., Pham V., Smith S., Chappell M., Breda L., Hurwitz S., Tanaka N., Castracani C.C. (2024). Effective Gene Therapy for Metachromatic Leukodystrophy Achieved with Minimal Lentiviral Genomic Integrations. bioRxiv.

[54] Yasuda E., Mackenzie W.G., Ruhnke K.D., Shimada T., Mason R.W., Zustin J., Martin P.L., Thacker M.M., Orii T., Sai Y. (2015). Long-Term Follow-Up of Post Hematopoietic Stem Cell Transplantation for Hurler Syndrome: Clinical, Biochemical, and Pathological Improvements. Mol. Genet. Metab. Rep..

[55] Selvanathan A., Ellaway C., Wilson C., Owens P., Shaw P.J., Bhattacharya K. (2018). Effectiveness of Early Hematopoietic Stem Cell Transplantation in Preventing Neurocognitive Decline in Mucopolysaccharidosis Type II: A Case Series. JIMD Rep..

[56] Snowden J.A., Sánchez-Ortega I., Corbacioglu S., Basak G.W., Chabannon C., de la Camara R., Dolstra H., Duarte R.F., Glass B., Greco R. (2022). European Society for Blood and Marrow Transplantation (EBMT). Indications for Haematopoietic Cell Transplantation for Haematological Diseases, Solid Tumours and Immune Disorders: Current Practice in Europe, 2022. Bone Marrow Transplant..

[57] Greco R., Ruggeri A., McLornan D.P., Snowden J.A., Alexander T., Angelucci E., Averbuch D., Bazarbachi A., Hazenberg M.D., Kalwak K. (2025). Indications for Haematopoietic Cell Transplantation and CAR-T for Haematological Diseases, Solid Tumours and Immune Disorders: 2025 EBMT Practice Recommendations. Bone Marrow Transplant..

[58] Chen J., Larochelle A., Fricker S., Bridger G., Dunbar C.E., Abkowitz J.L. (2006). Mobilization as a Preparative Regimen for Hematopoietic Stem Cell Transplantation. Blood.

[59] Ruutu T., Volin L., Beelen D.W., Trenschel R., Finke J., Schnitzler M., Holowiecki J., Giebel S., Markiewicz M., Uharek L. (2011). Reduced-Toxicity Conditioning with Treosulfan and Fludarabine in Allogeneic Hematopoietic Stem Cell Transplantation for Myelodysplastic Syndromes: Final Results of an International Prospective Phase II Trial. Haematologica.

[60] Kantulaeva A.K., Gutovskaya E.I., Laberko A.L., Radygina S.A., Kozlovskaya S.N., Livshits A.M., Shelikhova L.N., Balashov D.N., Maschan M.A. (2020). Hematopoietic Stem Cell Transplantation with TCRαβ+/CD19+ Graft Depletion for Hemophagocytic Lymphohistiocytosis. Pediatr. Hematol. Immunopathol..

[61] Ringdén O., Ringdén O., Hägglund H., Runde V., Basu O., Kroschinsky F., Stockschläder M., Potter M.N. (1998). Faster Engraftment of Peripheral Blood Progenitor Cells Compared to Bone Marrow from Unrelated Donors. Bone Marrow Transplant..

[62] Mehta R.S., Saliba R.M., Alsfeld L.C., Jorgensen J.L., Wang S.A., Anderlini P., Al-Atrash G., Bashir Q., Ciurea S.O., Hosing C.M. (2021). Bone Marrow versus Peripheral Blood Grafts for Haploidentical Hematopoietic Cell Transplantation with Post-Transplantation Cyclophosphamide. Transplant Cell Ther..

[63] Wang Y., Liu D., Liu K., Xu L., Zhang X., Han W., Chen H., Chen Y., Wang F., Wang J. (2013). Long-Term Follow-Up of Haploidentical Hematopoietic Stem Cell Transplantation without In Vitro T Cell Depletion for the Treatment of Leukemia. Cancer.

[64] Luznik L., O’Donnell P.V., Symons H.J., Chen A.R., Leffell M.S., Zahurak M., Gooley T.A., Piantadosi S., Kaup M., Ambinder R.F. (2008). HLA-Haploidentical Bone Marrow Transplantation for Hematologic Malignancies Using Nonmyeloablative Conditioning and High-Dose, Post-Transplantation Cyclophosphamide. Biol. Blood Marrow Transplant..

[65] Rujkijyanont P., Morris C., Kang G., Gan K., Hartford C., Triplett B., Dallas M., Srinivasan A., Shook D., Pillai A. (2013). Risk-Adapted Donor Lymphocyte Infusion Based on Chimerism and Donor Source in Pediatric Leukemia. Blood Cancer J..

[66] Mathews J.A., Borovsky D.T., Reid K.T., Murphy J.M., Colpitts S.J., Carreira A.S., Moya T.A., Chung D.C., Novitzky-Basso I., Mattsson J. (2024). Single Cell Profiling of Hematopoietic Stem Cell Transplant Recipients Reveals TGF-β1 and IL-2 Confer Immunoregulatory Functions to NK Cells. iScience.

[67] Chan Y.L.T., Zuo J., Inman C., Croft W., Begum J., Croudace J., Kinsella F., Maggs L., Nagra S., Nunnick J. (2018). NK Cells Produce High Levels of IL-10 Early after Allogeneic Stem Cell Transplantation and Suppress Development of Acute GVHD. Eur. J. Immunol..

[68] BMT CTN (2022). Protocol: CL-0304.

[69] Haines H.L., Bleesing J.J., Davies S.M., Hornung L., Jordan M.B., Marsh R.A., Filipovich A.H. (2015). Outcomes of Donor Lymphocyte Infusion for Treatment of Mixed Donor Chimerism after a Reduced-Intensity Preparative Regimen for Pediatric Patients with Nonmalignant Diseases. Biol. Blood Marrow Transplant..

[70] Pappas K.B. (2023). Newborn Screening. Pediatr. Clin. N. Am..

[71] Kelly N., Makarem D.C., Wasserstein M.P. (2016). Screening of newborns for disorders with high benefit–risk ratios should be mandatory. J. Law Med. Ethics.

